# Catalytic and Functional Roles of Conserved Amino Acids in the SET Domain of the *S. cerevisiae* Lysine Methyltransferase Set1

**DOI:** 10.1371/journal.pone.0057974

**Published:** 2013-03-01

**Authors:** Kelly Williamson, Victoria Schneider, Rachel A. Jordan, John E. Mueller, Michelle Henderson Pozzi, Mary Bryk

**Affiliations:** 1 Department of Biochemistry and Biophysics, Texas A&M University, College Station, Texas, United States of America; 2 Texas A&M University System Health Science Center, HSC College of Medicine, College Station, Texas, United States of America; 3 Lynntech, College Station, Texas, United States of America; St Jude Children’s Research Hospital, United States of America

## Abstract

In *S. cerevisiae*, the lysine methyltransferase Set1 is a member of the multiprotein complex COMPASS. Set1 catalyzes mono-, di- and trimethylation of the fourth residue, lysine 4, of histone H3 using methyl groups from S-adenosylmethionine, and requires a subset of COMPASS proteins for this activity. The methylation activity of COMPASS regulates gene expression and chromosome segregation *in vivo*. To improve understanding of the catalytic mechanism of Set1, single amino acid substitutions were made within the SET domain. These Set1 mutants were evaluated *in vivo* by determining the levels of K4-methylated H3, assaying the strength of gene silencing at the rDNA and using a genetic assessment of kinetochore function as a proxy for defects in Dam1 methylation. The findings indicate that no single conserved active site base is required for H3K4 methylation by Set1. Instead, our data suggest that a number of aromatic residues in the SET domain contribute to the formation of an active site that facilitates substrate binding and dictates product specificity. Further, the results suggest that the attributes of Set1 required for trimethylation of histone H3 are those required for Pol II gene silencing at the rDNA and kinetochore function.

## Introduction

Eukaryotic DNA is assembled into higher-order chromatin structures that promote compaction and protection of DNA. The structure of chromatin is dynamic to provide access to the underlying DNA template for nuclear processes, such as transcription and replication, and is controlled by several mechanisms [1]. Although the mechanisms of chromatin regulation by methylated histones are not as well understood as those governed by acetylated histones, a large body of work supports roles for methylated histones in the regulation of euchromatin and heterochromatin [2], [3], [4], [5].

Lysine-methylated histones can have diverse effects on transcription, ranging from regulation of Pol II initiation and elongation to the formation and maintenance of repressive heterochromatin [5], [6]. Histone methylation can be more complex than other covalent modifications because multiple methyl groups can be present at the same lysine residue that may alter the function of chromatin in different ways [2], [7]. Moreover, regulatory proteins can discriminate the different methylated forms of a histone, providing means to increase the types of signals presented by chromatin [8].

**Figure 1:**
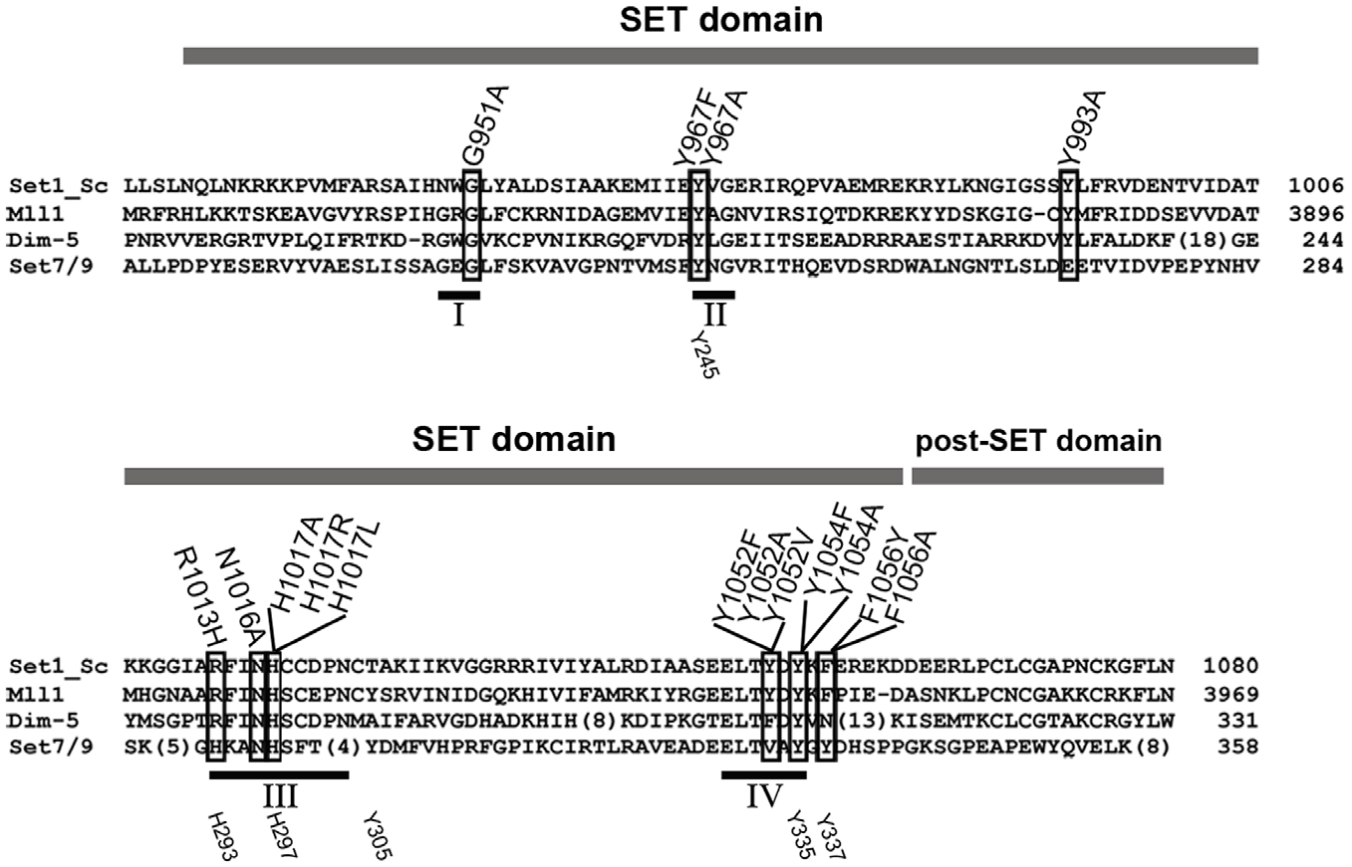
Alignment of SET domain methyltransferase proteins. Amino acid sequences of SET domain proteins from *S. cerevisiae* Set1 (Set1_Sc), *H. sapiens* MLL1, *N. crassa* Dim-5, and *H. sapiens* Set7/9 are aligned to show conserved residues. Gray bar above the aligned sequences indicates the SET and post-SET domains. Amino acid substitutions of Set1 analyzed here are indicated above and boxed within the alignment. The conserved SET motifs I-IV are shown below the alignment with black bars and Roman numerals. The numbers at the right end of the aligned sequences refer to the amino acid at the end of sequence. Numbers below the alignment (e.g. Y245, H293, etc.) indicate amino acids and their positions in Set7/9.

Most lysine methyltransferases (KMTases) contain a SET domain of ∼130 amino acids that is responsible for the catalysis of methyl group transfer from *S*-adenosylmethionine (AdoMet) to specific lysine residues within histone tails and other substrates [9], [10]. The SET domain has four conserved sequence motifs (Fig. 1) that support the methyl transfer reaction [11]. SET motif I contains a GxG motif that along with amino acid residues in SET motifs III (RFINHxCxPN) and IV (ELxFDY) interacts with the methyl donor AdoMet [12]. SET motif II has the amino acid sequence YxG with a tyrosine residue (Y) that has been hypothesized to act as an active site base in the SET domain methyl-transfer mechanism [13], [14]. In addition, SET motifs III and IV interact with each other forming a pseudoknot that contains the active site adjacent to the AdoMet and the target lysine binding sites [13], [14], [15].

SET domain KMTases vary with respect to product specificity, defined as the ability to transfer one, two, or three methyl groups to the target lysine [16]. These enzymes also vary in their ability to act independently or as a member of a multiprotein complex. Set7/9 is a human monomethyltransferase that acts independently to transfer one methyl group to lysine 4 of histone 3 (H3K4) [13], [17], [18]. Likewise, Dim5, a trimethyltransferase from *N. crassa*, acts independently to methylate H3K9 [14], [19]. Conversely, the KMTases Set1 from *S. cerevisiae* and MLL1 from *H. sapiens* catalyze mono-, di- and trimethylation of H3K4, and each functions as a member of a multiprotein complex [20], [21], [22], [23], [24].

Human SET domain KMTases Set7/9 and MLL1 have been the focus of structural studies. Crystal structures of Set7/9 have identified residues that contact the substrates AdoMet and the target lysine [13], [17], [18]. This structural information has been analyzed using molecular dynamics, hybrid quantum mechanics, molecular mechanics, and free-energy simulations to gain insights into the mechanism of Set7/9 [25], [26], [27]. *Mixed lineage leukemia protein-1* (MLL1) is named such because chromosomal translocations involving the MLL1 gene are associated with acute lymphoblastic and myelogenous leukemias [28], [29]. *In vivo*, the MLL1 multiprotein complex acts as a histone H3 trimethyltransferase [29], [30], [31] that regulates transcription [32], [33]. Recombinant expression and purification of MLL1 has allowed for analysis of its KMTase activity independently and as part of a minimal core complex. When assayed independently, MLL1 is a slow monomethyltransferase, but in the presence of a core complex of proteins, including Ash2L and RbBp5, MLL1 is a higher-order KMTase, indicating that other MLL1 complex members influence product specificity [20], [21], [23], [34], [35].

There has been some debate regarding the mechanism SET-domain proteins use to catalyze methyl transfers to lysine side chains [36]. In order for the S_N_2 methyl transfer reaction to occur, the target ε-amino group of lysine must be deprotonated. Two mechanisms for lysine deprotonation have been proposed, one involving deprotonation by an active site base and the second requiring deprotonation via an active site water channel.

Early studies on Set7/9 concluded that a conserved tyrosine residue in the SET domain behaves as an active site base to facilitate deprotonation of the target ε-amino group of lysine. However, two different tyrosines were identified as the potential active site base, one from the YxG SET motif II (Y = Tyr245) [17] and the other from the ELxFDY SET motif IV (Y = Tyr335) [17], [26]. In support of the proposed active site base mechanism, a structural study with Dim-5 showed that Tyr178 (equivalent to Tyr245 in Set7/9) interacts with the substrate lysine in a manner that would facilitate its deprotonation while a deprotonated Tyr283 (equivalent to Tyr335 in Set7/9) could stabilize the positively charged AdoMet [14]. However, as detailed below, recent work supports deprotonation via an active site water channel.

The purpose of a water channel in the SET domain active site is two-fold: (1) to promote hydrogen bonding within the active site to position important residues and substrates in the methyl transfer reaction and (2) to function as a proton acceptor for deprotonation of lysine [25], [37], [38]. In early modeling studies, when Tyr245 of Set7/9 was substituted with phenylalanine, the ε-amino group of the lysine substrate became exposed to a water channel [25], a result that is in agreement with other work showing that the Set7/9 Y245F mutant could catalyze higher-order methylation [13]. These modeling studies suggested that Tyr245 forms hydrogen-bonding interactions with a water channel and established the presence of water molecules within the active site of Set7/9 [13], [25]. Modeling studies with Set7/9 also indicated that the hydroxyl group of Tyr335, the conserved tyrosine residue in SET motif IV, has a higher calculated p*K_a_* than the ε-amino group of the lysine substrate containing zero, one, or two methyl groups, making it unlikely that Tyr335 would behave as an active site base [25], [38]. Recently, a crystallographic study with Set7/9 mutants using peptides bearing zero, one, two, and three methyl groups on the ε-amino group of the target lysine has provided insight into the role of water molecules in modulating multiple methylation events [37]. This study concluded that a water channel within the active site accepts the dissociated proton from the lysine substrate. Therefore, the active-site residues that form the access channel for the target lysine, including the conserved tyrosine residues in SET motifs II and IV, facilitate substrate binding and methyl transfer by creating a distinct volume that discriminates the methylation state of the substrate and thus governs product specificity.

The *S. cerevisiae SET1* gene encodes Set1, a member of the COMPASS complex that catalyzes methylation of lysine residues in histone H3 and the kinetochore-associated protein Dam1 [22], [39], [40], [41]. Set1 and K4-methylated H3 are required for silencing of Pol II transcription in the ribosomal DNA locus (rDNA) and at telomeres in *S. cerevisiae* [42], [43], [44], [45], [46]. Studies to identify catalytically important residues in the active site of Set1 have been few in number, most likely due to the inability to prepare active Set1 protein for *in vitro* structural and mechanistic studies. To overcome this limitation, we performed a mutational analysis of the SET domain of Set1 to gain insight into the mechanism of methyl transfer. Single amino acid substitution mutants of Set1 were characterized using *in vivo* assays, including histone H3 methylation and transcriptional silencing at the rDNA. In addition, a genetic suppression assay was used to indirectly assess methylation of the kinetochore protein Dam1 to gain insight into the possible role of conserved amino acid residues in methylation of a non-histone substrate [41]. Analysis of Set1 activity in *S. cerevisiae* cells is possible because there is only one H3K4 methyltransferase in yeast [39], compared to at least ten in mammalian cells [2]. Moreover, Set1 is a member of the COMPASS complex that is capable of catalyzing three methylation states at K4 of histone H3, and thus the analysis of mutants provides a way to determine how amino acid substitutions affect product specificity. The results provide insight into the mechanism of methyl transfer by Set1 and information on the role of higher-order H3K4 methylation in silent chromatin and kinetochore function.

## Materials and Methods

### Media

Yeast media used in these experiments have been described elsewhere [47]. YPADT is YPD media supplemented with 40 mg/L of adenine sulfate and 20 mg/L of L-tryptophan.

### Plasmids

Plasmid MBB251 contains an *Xho*I-*Sac*II fragment with the *SET1* ORF flanked by 436 bp upstream and 347 bp downstream. MBB251 was modified by the addition of a *Cla*1 site 9 bp downstream of the *SET1* stop codon to make MBB484. The *Xho*I-*Sac*II fragment of MBB484 was ligated into pRS406 to create MBB491, a vector for integration of *SET1* sequences into *ura3-52*. To create a mutagenesis shuttle vector, the pBluescript plasmid (Stratagene) was modified by cloning a linker containing a *Mun*I restriction site into the unique *Eco*RI site to create plasmid MBB479. Next, the *Mun*I-*Cla*I fragment of *SET1* from MBB484 was ligated into MBB479 creating MBB487.

### Mutagenesis

*SET1* mutants were generated by site-directed mutagenesis of MBB487 with Phusion polymerase (New England Biolabs) and specific primers (Table S2). Mutations were verified by DNA sequence analysis. The *Mun*I-*Cla*I fragment from each mutated plasmid was ligated into the *Mun*I-*Cla*I interval of MBB491.

### Yeast Strains

All *S. cerevisiae* strains are listed in Table S1. Yeast strains were generated by standard genetic crosses and transformation techniques. Wild-type and mutant alleles of *SET1* in the vector MBB491 and the empty vector itself were digested with *Stu*I and transformed into MBY2269 and MBY2450 (ZK2 *Δset1*, a gift from Sharon Dent). Integration of a single copy of MBB491 with wild-type *SET1* or derivatives containing mutant alleles of *SET1* into the *ura3-52* locus was verified by PCR and Southern blot analysis. The wild type and mutant *SET1* alleles integrated at *ura3-52* in MBY2269 derivatives were amplified from genomic DNA and each mutation was verified by DNA sequencing. MBY1198 with the *SET1* gene at its endogenous location, MBY2269 and derivatives containing wild-type or mutant alleles of *SET1* integrated at *ura3-52* were used in Western analyses and Northern analyses. Yeast strain MBY2450 (ZK2 *Δset1*) and its derivatives containing wild-type or mutant alleles of *SET1* were used in the *ipl1-2* growth experiments.

### Whole Cell Protein Extracts and Western Blot Analyses

Whole cell proteins were extracted as in [46]. Western blotting was performed, as described previously with some modifications [48]. For quantitative analysis of steady-state levels of K4-methylated histone H3, 8–80 µg of whole cell extract were resolved on 4–20% SDS-PAGE gradient gels (Thermo Fisher Scientific) and transferred to polyvinylidene fluoride (PVDF) membranes. Blots were incubated with primary antisera: anti-K4-monomethyl H3 (07-436, Upstate Cell Signaling or ab8895, Abcam; 1∶500), anti-K4-dimethyl (07-030, Upstate Cell Signaling; 1∶5000), anti-K4-trimethyl (ab8580, Abcam; 1∶5000), anti-histone H3 (ab1791, Abcam; 1∶1000) or anti-phosphoglycerate kinase (Pgk1, A6457, Molecular Probes; 1∶1000). After washing, blots were incubated with either HRP-conjugated anti-rabbit or anti-mouse secondary antibodies (Promega; 1∶2000–1∶5000 and 1∶2000, respectively), followed by treatment with Immuno-Star HRP substrate kit (Bio-Rad) to visualize specific proteins. Western blots were imaged and quantified using the Molecular Imager ChemiDoc XRS with Quantity One software (Bio-Rad). The average ratio of H3K4Me/total histone H3 (or Pgk1 protein) normalized to the Set1^+^ wild-type strain is shown in Table 1. The specificity of antisera against K4-methylated H3 was verified by performing peptide blots using unmodified and K4-methylated H3 peptides (data not shown).

**Table 1:** Summary of phenotypes of Set1^+^ cells, *set1Δ* cells and Set1 mutants.

Class^a^	Substitution^b^	*in vivo* methylation H3K4^c^			rDNA silencingTy1*his3AI/PYK1* d	Suppression of*ipl1-2* e
		K4me1	K4me2	K4me3		
null	Y967A	0.05+/−0.04	0	0	13.0+/−5.5, n = 6	Yes
null	N1016A	0	0	0	5.1+/−2.6, n = 6	Yes
null	H1017L	0.01+/−0.01	0	0.01+/−0.01	5.0+/−1.8, n = 7	Yes
null	H1017R	0.03+/−0.02	0.01	0	4.0+/−1.5, n = 4	Yes
null	Y1054A	0	0	0	3.3+/−0.7, n = 3	Yes
null	F1056A	0	0	0	6.8+/−2.8, n = 7	Yes
partial func/null	G951A	0.70+/−0.14	0.02+/−0.01	0	8.1+/−3.9, n = 6	partial-yes
partial func/null	Y993A	0.49+/−0.06	0.06+/−0.04	0	8.0+/−3.2, n = 5	partial-yes
partial func/silent	Y967F	0.50+/−0.05	0.01	0.04+/−0.03	2.0+/−1.0, n = 6	partial-no
partial func/silent	R1013H	1.33+/−0.02	0.54+/−0.05	0.02+/−0.01	2.8+/−0.3, n = 3	partial-no
partial func/silent	Y1054F	0.45+/−0.07	0.28+/−0.04	0.03+/−0.01	1.7+/−0.2, n = 3	partial-no
silent	H1017A	1.52+/−0.14	1.98+/−0.30	3.18+/−1.46	1.0+/−0.3, n = 4	No
silent	Y1052F	0.56+/−0.02	0.84+/−0.16	1.49+/−0.33	1.0+/−0.1, n = 6	No
silent	Y1052A	0.35+/−0.16	0.59+/−0.11	1.05+/−0.23	0.8+/−0.2, n = 6	No
silent	Y1052V	0.63+/−0.20	1.24+/−0.30	2.70+/−0.74	1.0+/−0.3, n = 6	No
silent	F1056Y	0.96+/−0.06	0.92+/−0.17	1.20+/−0.50	0.8+/−0.1, n = 3	No
Wild type Set1^+^	None	1	1	1	1	No
*set1Δ*	deletion	0	0	0	5.4+/−1.7, n = 30	Yes

a For details of classification, see text; partial func/null, partial function with phenotypes more similar to *set1Δ* cells; partial func/silent, partial function with phenotypes more similar to Set1^+^ cells.

b Amino acid substitution in Set1.

c Average levels +/− range of H3K4me1, H3K4me2 and H3K4me3 measured in whole cell extracts from Set1 mutants by quantitative western blotting (n = 2); values are normalized to the levels measured in whole cell extracts from a wild type Set1^+^ strain. No value for +/− range is given if the two measurements were identical. See Figure 3 and text for details.

d Pol II gene silencing at the rDNA was assessed by measuring the level of Ty1*his3AI* mRNA/*PYK1* mRNA in total RNA relative to that from a Set1^+^ strain where the ratio of Ty1*his3AI*/*PYK1* was set to 1. The values shown are average +/− SD for n repeats.

e Suppression of *ipl1-2* growth defects at the restrictive temperature (30°C) was measured in growth assays described in the text and shown in Figure 5. Yes, growth at 30°C; No, severely reduced growth at 30°C; partial-yes, slightly reduced growth at 30°C; partial-no, intermediate reduction of growth at 30°C.

To measure steady-state levels of Set1 protein, 150 µg of whole cell extract were resolved on 7% SDS-PAGE gels and transferred to PVDF membranes. Blots were incubated with anti−Set1 (sc-25945, Santa Cruz Biotechnology; 1∶1000), washed and then incubated with HRP-conjugated anti-goat secondary antibodies (sc-2020, Santa Cruz Biotechnology; 1∶1000). After washing, blots were developed and imaged as described above, and then stained with Ponceau S to visualize total protein and serve as a loading control [49].

### Northern Blot Analysis

Isolation of total RNA and Northern blotting were performed as described previously [42], [50]. Strand-specific ^32^P -labeled RNA probes or DNA probes were used to detect Ty1*his3AI* and *PYK1* mRNAs [51]. Northern blots were imaged with a Pharos FX Plus Molecular Imager and quantified using Quantity One software (Bio-Rad).

### Plate Growth Assay Using ipl1-2 Strains

Strains containing *ipl1-2* were grown in 5 mL of YPADT to saturation at 25°C. Ten-fold serial dilutions were made in sterile water and equal volumes (4 µl) of each dilution were spotted onto three YPADT agarose plates. Plates were incubated at 25°C, 30°C or 37°C for 1–2 days prior to imaging.

## Results

### Identification of Conserved Residues within the SET Domain of Set1

The Set1 sequence was aligned with other KMTases to identify the four conserved SET domain motifs and the locations of conserved amino acids that may play an important role in protein methylation. Based on the sequence alignment and structural data from Set7/9, DIM-5 and MLL1 [11], [13], [14], [20], point mutations were made at one or more residues in each of the conserved motifs in Set1 (Fig. 1).

### Expression of Set1 Mutants

**Figure 2:**
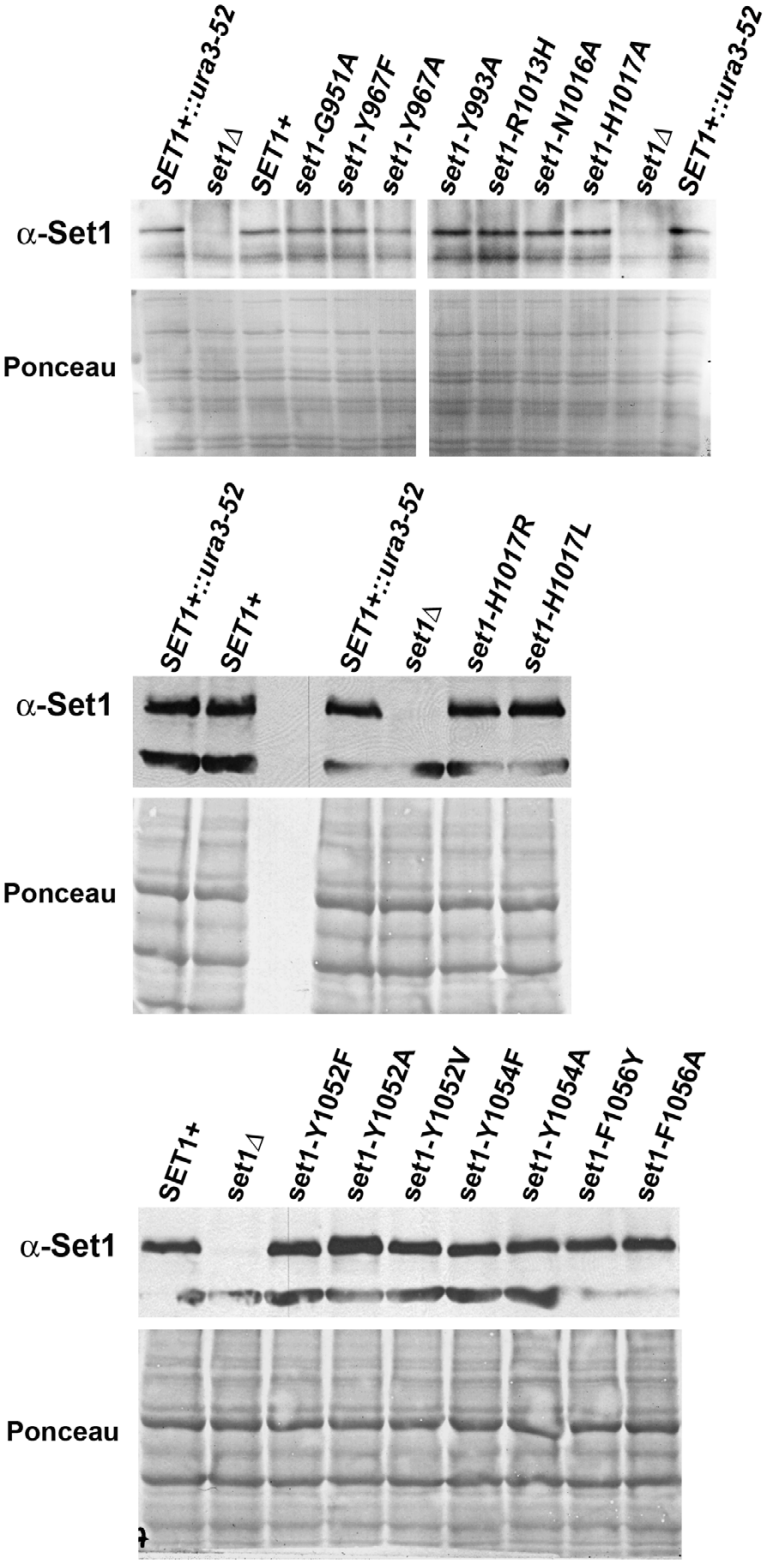
Cells containing wild-type or mutant alleles of *SET1* at *ura3-52* express similar steady-state levels of Set1 protein. Whole cell extracts (150 µg) from wild type Set1^+^ cells (with the *SET1* gene at its endogenous location, *SET1+*, or integrated at the *ura3-52* locus, *SET1+::ura3-52*), Set1 deletion cells (*set1Δ*) and Set1 mutants (indicated above each panel) were separated and transferred to PVDF membranes. For each blot, the upper panel is an immunoblot showing the steady-state level of Set1 protein in whole cell extracts; the lower panel is the same membrane stained with Ponceau S to verify equal loading of protein extracts. The dark band below the Set1 band in the immunoblots is a non-specific band. Representative data are shown (n≥3).

Each *SET1* mutant allele was integrated into the *ura3-52* locus of a *S. cerevisiae* strain lacking the endogenous *SET1* gene (Table S1). A strain with the *SET1* gene at its endogenous locus as well as strains either lacking *SET1* or containing a wild-type copy of *SET1* integrated at the *ura3-52* locus were analyzed as controls. To verify that cells with the wild type or a mutant *SET1* gene integrated at *ura3-52* express stable Set1 protein, Western blotting assays were performed using whole cell extracts and antibodies specific for Set1 (Fig. 2). The results show that the steady-state level of Set1 protein was similar in protein extracts from wild-type cells (*SET1^+^* and *SET1^+^::ura3-52*) and each of the sixteen amino acid substitution mutants. In contrast, background signal was detected in extracts from *set1Δ* cells that lack Set1 protein.

### Steady-state Levels of K4-methylated Histone H3 in the Set1 Mutants

**Figure 3:**
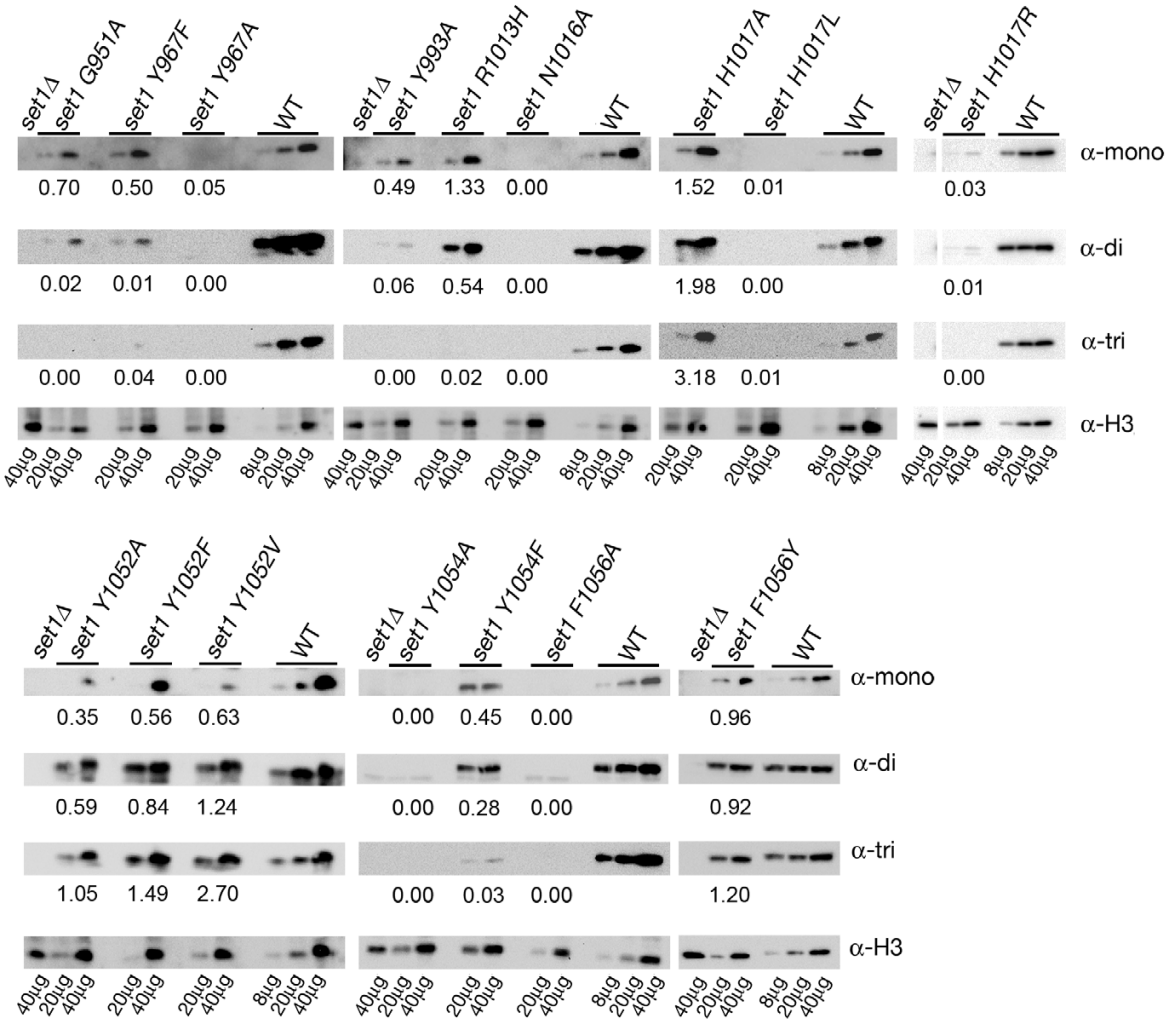
Quantitative Western blots measuring steady-state levels of K4-methylated histone H3 in cells expressing wild-type and mutant alleles of *SET1.* Representative Western blotting experiments are shown for cells carrying wild-type *SET1* at its endogenous location, *set1Δ* and *set1* amino acid substitution alleles. Dilutions of protein extracts (µg loaded indicated below the lower panel) from wild-type (WT), *set1Δ,* and Set1 mutant cells were analyzed by Western blotting with specific antibodies to measure the *in vivo* steady-state levels of K4-monomethylated H3 (α-mono), K4-dimethylated H3 (α-di), and K4-trimethylated H3 (α-tri). The level of histone H3 (α-H3) or Pgk1 (α-Pgk1) protein was used to normalize the amount of protein loaded in each lane. The average level of normalized K4-methylated H3 detected in Set1 mutant extracts relative to wild-type extracts is shown below each blot (n = 2). See Table 1 for the normalized average +/− range for each mutant.

Western blotting experiments using whole cell protein extracts were conducted to assess the ability of the Set1 mutants to methylate K4 of histone H3 (Fig. 3, Table 1). Specific antisera were used to detect the steady-state level of K4-monomethylated histone H3 (H3K4me1), K4-dimethylated histone H3 (H3K4me2) or K4-trimethylated histone H3 (H3K4me3). Control extracts from *set1Δ* cells lacked detectable K4-methylated H3, verifying that Set1 is the only K4-specific histone H3 methyltransferase in *S. cerevisiae* [39]. A range of levels of the three forms of K4-methylated H3 was detected in whole cell extracts from the Set1 mutants. Classification of some mutants was clear based on the steady-state levels of K4-methylated H3. For example, certain Set1 SET domain mutants behaved like *set1* null mutants with extremely low or undetectable levels of the three forms of K4-methylated H3 (Y967A, N1016A, H1017L, H1017R, Y1054A and F1056A). Other Set1 mutants had steady-state levels of K4-methylated H3 that were variable with one or more forms being higher than the levels measured in extracts from wild type Set1^+^ cells (R1013H, H1017A, Y1052F, Y1052A, Y1052V and F1056Y). Three of the mutants (G951A, Y967F and Y993A) had levels of H3K4me1 at ∼50–70% of wild type but the levels of H3K4me2 and H3K4me3 were 6% of wild type or lower. In the remaining mutant Y1054F, the levels of the three forms of K4-methylated H3 were less than 50% of wild type. To learn more about the function of these Set1 mutants in gene silencing at the rDNA and kinetochore function, additional assays of Set1 function were performed.

### Gene Silencing Activity of Set1 Mutants

**Figure 4:**
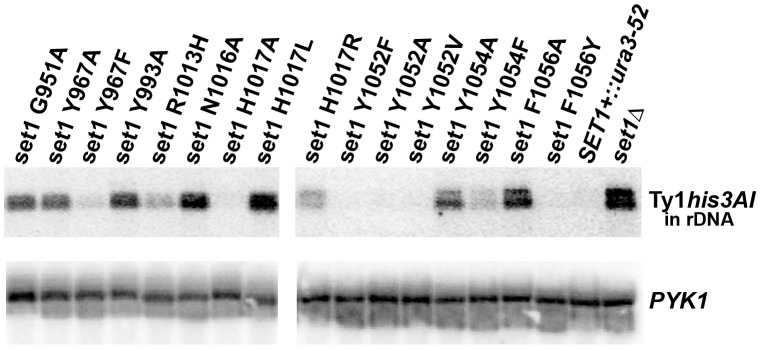
Northern blot analysis to evaluate gene silencing at the rDNA. Representative Northern blots are shown for cells carrying wild-type *SET1*, *set1Δ* and *set1* amino acid substitution mutants. The source of total RNA is indicated above the top panel for each set of blots. Ty1*his3AI*, a Pol II-transcribed gene inserted in the rDNA locus, was used to assess gene silencing at the rDNA. *PYK1* mRNA was used to normalize the amount of RNA loaded in each lane. See Table 1 for the normalized average +/− standard deviation for each mutant. Each mutant was analyzed in 3 or more independent experiments. Total Ty1 mRNA was measured in each sample to verify that mutation of Set1 did not increase the level of total Ty1 mRNA in the cells (data not shown).

Previous work has shown that Pol II-dependent expression of a Ty1*his3AI* element in the rDNA is repressed by rDNA-specific silent chromatin that requires Set1 [39], [42], [50]. The steady-state level of mRNA from the Ty1*his3AI* element in the rDNA was measured in total RNA from cells lacking Set1 (*set1Δ*), expressing wild-type Set1 or expressing one of the Set1 mutants, to evaluate the ability of the Set1 mutants to support rDNA silencing of Pol II transcription. *PYK1* mRNA was measured to normalize the amount of RNA loaded in each lane. Representative results are shown in Figure 4. The mean value of the ratio of Ty1*his3AI* mRNA to *PYK1* mRNA with standard deviation for each Set1 mutant normalized to the ratio for cells expressing wild-type Set1 are given in Table 1.

In *set1Δ* cells, the steady-state level of Ty1*his3AI* transcript was increased 5.4-fold (Fig. 4, Table 1) compared to wild type Set1^+^ cells, consistent with the loss of rDNA silencing of Pol II transcription [39], [42]. For most of the Set1 mutants, the ability of a mutant Set1 protein to maintain Pol II gene silencing at the rDNA correlated directly with its ability to methylate histone H3 *in vivo* (Figs. 3 and 4; Table 1). Several mutants (Y967A, N1016A, H1017L, H1017R, Y1054A, and F1056A) with low or undetectable levels of all three forms of K4-methylated histone H3 exhibited defects in Pol II gene silencing, as expected.

Most Set1 mutants with levels of one or more forms of K4-methylated H3 greater than wild type (H1017A, Y1052F, Y1052A, Y1052V and F1056Y) retained the ability to silence the Ty1*his3AI* gene in the rDNA. In these mutants, the level of Ty1*his3AI* mRNA was similar to the level observed in wild type Set1^+^ cells (Table 1). However, the silencing phenotype of the R1013H mutant separated it from others in this group. Despite having a level of H3K4me1 that was higher than that in wild-type cells, the R1013H mutant was defective for gene silencing at the rDNA with a steady-state level of Ty1*his3AI* mRNA that was 2.8-fold higher than the level in wild type Set1^+^ cells. Notably, the R1013H mutant had a considerably reduced level of H3K4me3 (2% of wild type) compared to the H1017A, Y1052F, Y1052A, Y1052V and F1056Y mutants.

The results from the four remaining Set1 mutants suggest a correlation between silencing and H3K4me3. Specifically, the Set1 mutants G951A and Y993A were defective for Pol II gene silencing at the rDNA with average ratios of Ty1*his3AI*/*PYK1* mRNA that were increased approximately eight-fold compared to wild type Set1^+^ cells (Fig. 4; Table 1). In contrast, the Y967F mutant had an average ratio of Ty1*his3AI*/*PYK1* mRNA that was two-fold higher than the wild-type strain but the difference was not statistically significant. Given the similarities in the levels of H3K4me1 and H3K4me2 in these three mutants (Fig. 3 and Table 1), one possibility is that the low level of H3K4me3 detected in the Y967F mutant (4% of wild type) is sufficient to support rDNA silencing, while the lower levels of H3K4me3 in G951A and Y993A mutants (undetectable) are not. The Set1 mutant Y1054F also exhibited defects in gene silencing at the rDNA, though less severe than other mutants (1.7 fold increase in Ty1*his3AI*/*PYK1*). It is interesting to note that, like the R1013H mutant with a less severe rDNA-silencing defect (2.8-fold compared to WT), a low level of H3K4me3 was also detected in extracts from the Y1054F mutant (Fig. 3; Table 1). The levels of H3K4me3 in these mutants (Y967F, 4%, R1013H, 2% and Y1054F, 3%) suggest that a relatively low steady-state level of H3K4me3 is sufficient to support Pol II gene silencing at the rDNA.

### Suppression of the *ipl1-2* Mutation by the Set1 Mutants as a Proxy for Methylation of a Non-histone Substrate, Dam1

**Figure 5:**
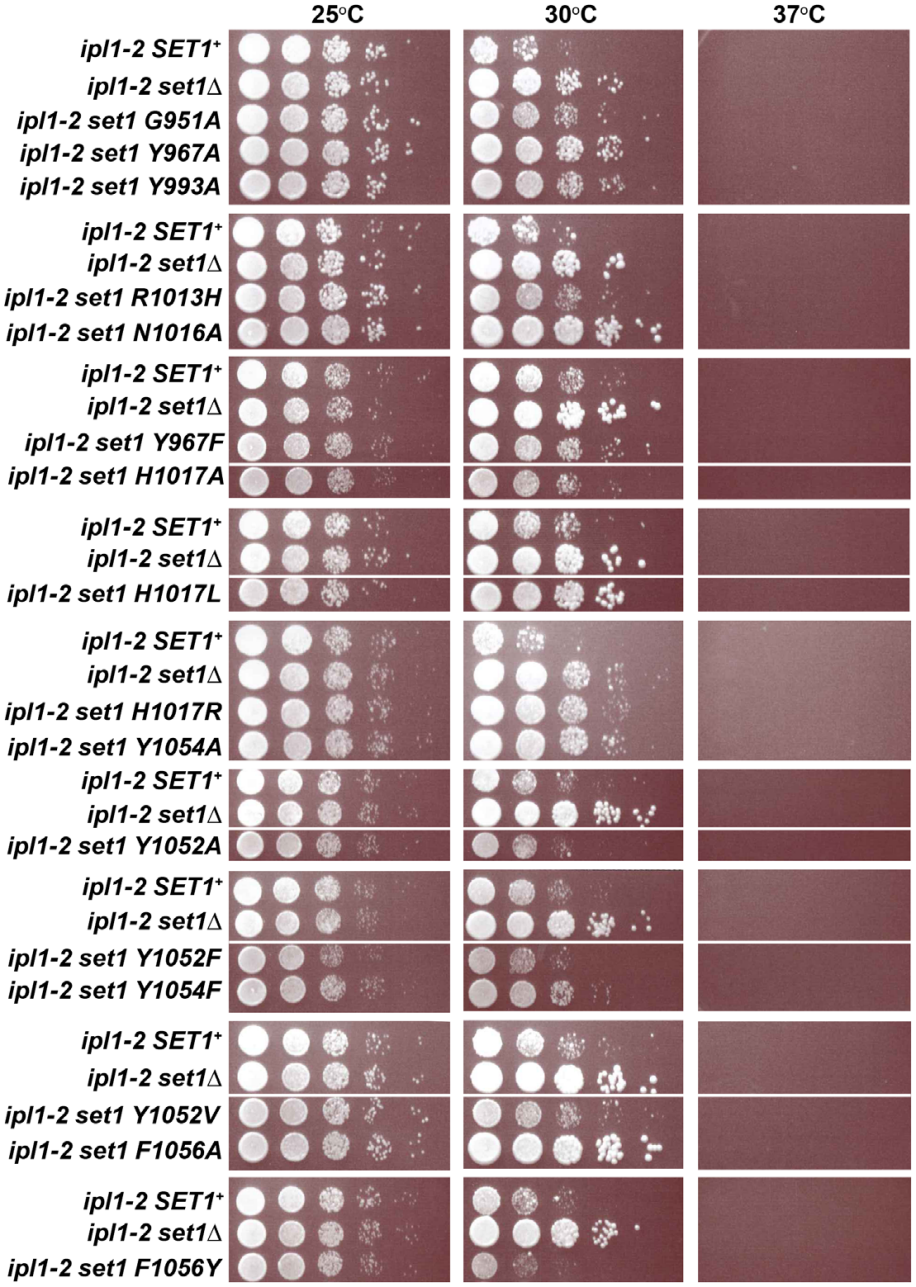
Suppression of *ipl1-2* by Set1 mutants. The ability of the Set1 amino acid substitution mutants to suppress the temperature sensitive growth phenotype of *ipl1-2* mutants at 30°C was tested using plate growth assays. Dilutions (1∶10) of cultures of *ipl1-2 SET1^+^*, *ipl1-2 set1Δ* and *ipl1-2* Set1 amino acid substitution mutants were spotted on YPADT plates and incubated at 25°C, 30°C and 37°C for 24–48 hours. The ability of the Set1 mutants to suppress the *ipl1-2* growth defect at 30°C is summarized in Table 1.

Dam1, a member of the Dam1 complex that connects microtubules to the kinetochore and promotes proper chromosome segregation [52], [53], [54], is a non-histone substrate of Set1 [41]. A balance between methylation of Dam1 by Set1 and phosphorylation of Dam1 by the Aurora kinase Ipl1 is required for proper chromosome segregation and cell viability [41], [55], [56]. Deletion of *SET1* suppresses the temperature-sensitive growth defect of cells carrying a conditional allele of the Aurora kinase, *ipl1-2*. Previous work has shown that this suppression is due to reduced methylation of Dam1 in the absence of Set1 [41]. We tested the ability of the Set1 mutants to suppress the growth defects of *ipl1-2* cells at a restrictive temperature as an indirect test of their ability to methylate Dam1. Individual alleles encoding the Set1 amino acid substitution mutants were introduced into cells carrying an *ipl1-2* mutation, and the ability of the Set1 mutants to suppress the *ipl1-2* growth defect at a restrictive temperature (30°C) was tested (Fig. 5). Cells with a wild-type copy of the *SET1* gene (*ipl1-2 SET1^+^*) and those lacking *SET1* (*ipl1-2 set1Δ*) grew equally well at the permissive temperature (25°C). However, the *ipl1-2 SET1^+^* cells grew poorly at the restrictive temperature, 30°C. In contrast, the *ipl1-2 set1Δ* cells grew well, presumably because methylation of Dam1, and inappropriate chromosome attachment, which reduces cell viability, does not occur in the *set1Δ* cells at the restrictive temperature.

The results of growth assays measuring suppression of the *ipl1-2* conditional allele by the Set1 mutants are shown in Figure 5 and summarized in Table 1. Several mutants, Y967A, N1016A, H1017L, H1017R, Y1054A, and F1056A, with reduced or undetectable levels of K4-methylated histone H3, suppressed the growth defect of *ipl1-2* cells at 30°C, hinting that these Set1 mutants are likely to have lost the ability to methylate Dam1. In contrast, with the exception of the R1013H mutant, the Set1 mutants with levels of one or more forms of K4-methylated H3 greater than wild type (H1017A, Y1052A, Y1052F, Y1052V and F1056Y) did not suppress the *ipl1-2* growth defect at 30°C, suggesting that these Set1 mutants are likely to have retained the ability to methylate Dam1. The remaining Set1 mutants, G951A, Y967F, Y993A, R1013H and Y1054F, displayed partial suppression phenotypes, as evidenced by growth at the restrictive temperature that was intermediate between that of the *SET1^+^* and *set1Δ* cultures.

Based on the results shown in Figures 2, 3, 4, and 5, the Set1 mutants were classified based on their ability to methylate histone H3 and perform other functions *in vivo* (Table 1). One class of mutants was categorized as ‘null mutants’ due to the absence or low levels of K4-methylated H3 in whole cell extracts, the inability to silence expression of the Ty1*his3AI* element in the rDNA and the ability to suppress *ipl1-2* growth defects. The Set1 null mutants are Y967A, N1016A, H1017L, H1017R, Y1054A and F1056A. Because Set1 protein was expressed in these mutants (Fig. 2), we conclude that each of the amino acid substitutions abolish or significantly reduce methyl transfer activity *in vivo* without affecting the steady-state levels of the Set1 protein.

A second class contains five Set1 mutants with phenotypes that are similar to wild type Set1^+^ cells. The Set1 mutants in the ‘silent’ class are H1017A, Y1052F, Y1052A, Y1052V and F1056Y. These Set1 mutants maintained the ability to silence the Ty1*his3AI* element at the rDNA and failed to suppress the growth defects in cells with an *ipl1-2* mutation. Thus, these mutants are classified as silent with respect to rDNA silencing and suppression of the *ipl1-2* phenotype. A high degree of variability in the steady-state levels of the three forms of K4-methylated H3 was detected in this class of mutants, a result that suggests that small (≤3-fold) increases or decreases in methyl transfer activity do not interfere significantly with the function of Set1 in rDNA silencing and at the kinetochore.

A third class of mutants, the partial function mutants, has five members (G951A, Y967F, Y993A, R1013H and Y1054F). These mutants share the characteristic that each partially suppresses the growth defects of cells carrying the *ipl1-2* mutation at the restrictive temperature. Notably, these mutants exhibited broad differences in their steady-state levels of K4-methylated H3 and in their ability to silence Pol II transcription at the rDNA. Based on the severity of the rDNA-silencing phenotypes and *ipl1-2* suppression, the partial function Set1 mutants were divided into two subclasses. The partial function/null mutants, G951A and Y993A, exhibited a strong loss of rDNA silencing (Fig. 4, Table 1) and an *ipl1-2* suppression phenotype (Fig. 5) that was more similar to *set1Δ* cells than Set1^+^ cells. On the other hand, the partial function/silent mutants had phenotypes that were more similar to Set1^+^ cells (Table 1, Figs. 4 and 5). Implications of these data with respect to catalysis, rDNA silencing and suppression of the *ipl1-2* mutation are discussed below.

## Discussion

The covalent modification of proteins and the generation of the histone code play a central role in chromatin structure and function. In this study, we have analyzed several Set1 mutants to evaluate histone H3 methylation patterns and *in vivo* phenotypes associated with alteration or loss of Set1 function. Our results provide insights into the roles of specific residues of Set1 in catalysis of the three levels of H3K4 methylation. In addition, these mutants provide information to better understand the relationship between the methylation activity of Set1 and gene silencing at the rDNA locus of *S. cerevisiae*.

Although structural data for Set1 do not exist at this time, sequence alignments comparing Set1 to other SET-domain proteins identified conserved residues with the potential to play important roles in methylation reactions. For this study, mutants were generated with amino acid substitutions in one of the four conserved motifs of the SET-domain in *SET1*. For each mutant, the steady-state level of the mutant Set1 protein was found to be comparable to the level of Set1 protein in cells expressing wild-type *SET1* (Fig. 2). These results suggest that the amino acid substitutions do not affect the integrity of the Set1 protein. Therefore, the loss of methylation activity observed in these mutants is unlikely to be due to the loss of Set1 protein from the cell and therefore is likely to reflect changes in the methylation behavior of the Set1 mutant due to the substitution of a specific amino acid residue.

### Insights into the Catalytic Roles of Conserved Amino Acids in the SET Domain of Set1

Characterization of the methylation activity in five series of Set1 mutants, Y967F/A, H1017A/L/R, Y1052F/A/V, Y1054F/A, and F1056Y/A has provided information that distinguish between the two possible mechanisms of proton abstraction from the ε-amino group of the substrate lysine residue. As previously discussed, one mechanism proposes that deprotonation occurs due to an active site base and the second that deprotonation occurs via a water channel.

Set1 residues Y967 located in SET motif II and Y1054 located in SET motif IV align with conserved tyrosine residues in other SET-domain proteins, including Y178 in Dim-5 (Y967 in Set1) [14], and Y245 and Y335 in Set7/9 (Y967 and Y1054 in Set1, respectively) [17], [26] (Fig. 1). In the Dim-5 and Set7/9 structures, each of these tyrosine residues is in proximity to the ε-amino group of the target lysine such that each could potentially function as a general base to abstract a proton [14], [17], [26]. Our results indicate that the Y967A and Y1054A Set1 mutants lack methylation activity with H3K4 substrates. However, data from the Set1 mutants Y967F and Y1054F show that despite the loss of a hydroxyl group at this position, these mutants catalyze methylation reactions, albeit at reduced levels compared to wild type. The Set1 mutant Y967F has levels of H3K4me1 that are ∼50% of wild type and greatly reduced levels of H3K4me2 (1%) and H3K4me3 (4%) (Fig. 3; Table 1). The Set1 mutant Y1054F catalyzes methylation reactions producing 45% H3K4me1, 28% H3K4me2, and 3% H3K4me3 compared to wild-type Set1. The presence of K4-methylated H3 in cells with the Y967F and Y1054F Set1 mutants indicates that neither of these tyrosine residues is an essential active site base.

Our data suggest that Y967 and Y1054 in Set1 facilitate higher order H3K4 methylation reactions. This idea, supported by our data, is in agreement with structural data on Set7/9. In Set7/9, residues Y245 and Y335 (Y967 and Y1054 in Set1, respectively) contribute to the formation of the lysine access channel that forms the active site [13], contribute to a hydrogen bond network with other active-site residues [13], [25], [26], interact with water molecules that play a role in orienting the ε-amino group of the substrate lysine, and determine if multiple methyl transfer reactions can be catalyzed [27], [37], [57]. It has been proposed that mutating these conserved tyrosine residues in Set7/9 would cause the displacement of critical water molecules, thereby altering the active site in a way that would change product specificity [25]. Our results from the analysis of Set1 mutants at Y967 and Y1054 are in agreement with a model that these residues contribute to the formation of a hydrogen-bonding network that stabilizes the active site and promotes deprotonation to allow higher order methylation reactions. Whether these hydrogen bonds are with water molecules, other Set1 active site residues, or with the substrate lysine itself remains unknown.

There are several conserved aromatic residues in SET domain proteins (Fig. 1) that contribute to protein methylation. Two such residues in Set1 are Y1052 and F1056. To assess the role of Y1052, a series of mutants containing single amino acid substitutions were made, Y1052F, Y1052A, and Y1052V. This position in SET-domain proteins is referred to as the “Phe/Tyr switch” and has been shown to play a role in dictating product specificity [58]. According to the “Phe/Tyr switch” hypothesis, a tyrosine residue at this position prevents higher-order methylation explaining why tyrosine is usually found at this position in the SET-domain monomethyltransferases, whereas a phenylalanine residue at this position, which is the case in several higher-order methyltransferases, allows for multiple methyl additions. However, Set1, a higher-order methyltransferase, has a tyrosine residue at this location, Y1052, suggesting that this residue does not dictate product specificity in all SET domain proteins. Nonetheless, our results are in agreement with previous work showing that a Set1 Y1052F mutant produces greater trimethyltransferase activity [58].

Like *S. cerevisiae* Set1, the human MLL1 complex catalyzes H3K4-mono-, di-, and trimethylation despite having a tyrosine at the “Phe/Tyr switch” position. A mechanistic study indicated that purified MLL1 SET domain is a monomethyltransferase in the absence of other MLL1 complex members [21]. Studies using the MLL1 SET domain mutant Y3942F revealed that this amino acid substitution allowed the mutant protein to catalyze K4-mono-, di- and trimethylation of an H3 peptide [21]. Our Set1 mutants at the corresponding position, Y1052F/A/V, all had higher steady-state levels of H3K4me3 and reduced levels of H3K4me1 compared to wild-type Set1. These results are in agreement with a model that the residue at the “Phe/Tyr switch” position contributes to product specificity.

The other partially conserved aromatic residue F1056 in Set1 aligns with Y337 of Set7/9, a residue shown in crystal structures to form the active-site channel for the target lysine substrate [13], [17]. The Set1 mutants F1056Y and F1056A were constructed to investigate the importance of an aromatic residue at this position in the active site. Set1 F1056Y had nearly wild-type levels of the three forms of K4-methylated H3, maintained the *SET1* rDNA silencing phenotype and failed to suppress the conditional growth defect associated with the *ipl1-2* allele (Table 1). In contrast, Set1 F1056A is a null mutant, lacking the ability to catalyze methylation of H3K4 and able to suppress the *ipl1-2* growth defect (Table 1). Our results support a model that the aromatic residues Y1052 and F1056 play an essential role in formation of the active site channel allowing binding of the methyl acceptor, H3K4, and allowing multiple methylation events to occur at the target lysine ε-amino group.

A highly conserved histidine residue, H1017, is found within SET motif III of Set1 (Fig. 1). The corresponding residue H297 of Set7/9 was implicated in positioning of a conserved tyrosine residue (Y335 in Set7/9, Y1054 in Set1) required for the formation of the lysine access channel as well as participating in a hydrogen bond network [13], [17], [18]. A recent study with a H3907A MLL1 mutant, which corresponds to the H1017 position of Set1, has shown that this substitution mutant causes an AdoMet binding deficiency that was partially rescued when the Ash2L/RbBp5 heterodimer is associated with the H3907A MLL1 mutant [34]. In our study, three different Set1 mutants were made at H1017, each containing a single amino acid substitution, H1017A/L/R. The Set1 mutant H1017A has a hyper-methylation phenotype, catalyzing the formation of H3K4me1, H3K4me2, and H3K4me3 at levels that are at least 50% higher than those observed in wild type Set1^+^ cells (Table 1). The two other mutants, H1017R and H1017L, are null mutants despite the fact that the substituted residues maintain a similar van der Waals volume (Leu and Arg compared to His) and a positive charge (Arg compared to His). While no definitive conclusion about the role of H1017 can be made, our results with the H1017A mutant show that the size and charge of this residue can be altered without decreasing catalysis.

With respect to providing insight into the catalytic mechanism of Set1, our results indicate that no single active site base is required for methylation of H3K4. Instead, the data suggest that the conserved aromatic residues are critical for forming an active site that facilitates productive substrate binding and dictates product specificity. Cumulatively, these results are consistent with Set1 having a water channel in the active site that allows for deprotonation of the target lysine side chain and accommodation of increasing numbers of methyl groups covalently attached to the lysine ε-amino group.

### Insights into the Functional Roles of Set1 in vivo

The Set1 mutants were divided into three classes based on the effect of the mutation on Set1’s ability to catalyze methylation of H3K4, silence expression of a Pol II gene at the rDNA and to genetically interact with *ipl1-2*, a proxy for methylation of Dam1, a non-histone substrate (Table 1). The null class of mutants lacked the ability to methylate histone H3 on K4. Based on these results, we conclude that the amino acid substitutions do not support the methylation activity of Set1. This conclusion is supported by our results showing that each null mutant suppressed the *ipl1-2* conditional growth phenotype, a phenotype associated with a defect in methylation of Dam1 by Set1 (41). A second class of Set1 mutants had levels of K4-methylated H3 in whole cell extracts that were 50% or higher than the levels measured in extracts from wild type Set1^+^ cells. None of these mutants suppressed the growth defect of *ipl1-2* cells, suggesting that Set1 activity was not limiting for proper kinetochore function in these mutants. The partial function class of Set1 mutants retained some methylation activity with histone H3. These Set1 mutants provide insight into the role of methylation in rDNA silencing.

The Set1 ‘partial function mutants’ suggest that H3K4me3 is required for silencing of Pol II transcription at the rDNA (Fig. 4, Table 1). The Set1 mutants G951A and Y993A, which have slightly reduced levels of H3K4me1 and low or no H3K4me2 and H3K4me3, are defective for rDNA silencing, suggesting that either H3K4me2 or H3K4me3 is required for rDNA silencing. The Set1 mutants R1013H and Y1054F have wild type or slightly reduced levels of H3K4me1 and H3K4me2 and greatly reduced levels of H3K4me3. These mutants are also defective for rDNA silencing, although the phenotypes are not as severe as in the null mutants that fail to produce H3K4me3. Taken together, the silencing phenotypes of these four mutants suggest that the level of H3K4me3 is correlated directly with Pol II silencing in the rDNA. Interestingly, the results from the Y967F mutant indicate that a level of H3K4me3 that is ∼4% of that found in wild-type cells represses Pol II transcription at the rDNA in a manner that is not significantly different from that observed in wild type Set1^+^ cells (Y967F, *Ty1his3AI* mRNA/*PYK1* mRNA, 2.0+/−1.0, n = 6; Table 1). A requirement for H3K4me3 in rDNA silencing is consistent with a conclusion from previous work using Set1 mutants lacking portions of the N-terminus of the protein that resulted in H3K4me3-deficient cells that were defective in rDNA silencing [43]. However, this conclusion was based on the function of a truncated Set1 protein that could have altered COMPASS complex assembly [59]. Our data from the partial function Set1 mutants indicate that the level of H3K4me3 in a cell is related to silencing capacity of rDNA chromatin. Whether K4-trimethylated H3 is required directly at the rDNA locus or if it regulates silent chromatin indirectly remains unknown.

Our results suggest that Set1 trimethylation activity, rDNA silencing, and *ipl1-2* interaction are correlated, indicating that while Set1 promotes multiple types of methylation, these Set1 phenotypes relate specifically to its trimethylation activity. For example, analysis of the ability of the Set1 mutants to suppress the growth defect of cells with a conditional allele of *S. cerevisiae* gene encoding Aurora kinase, *ipl1-2*, has revealed structural aspects of the active site of Set1 that promote normal kinetochore function. The Set1 mutants that retained the ability to generate H3K4me3, even at levels below 10% of wild type, failed to suppress the growth defect of *ipl1-2* cells (Figs. 3, 5 and Table 1). These results suggest that features of the Set1 active site that are required for trimethylation of histone H3 may also be required for methylation of Dam1.

Efforts to express and purify recombinant Set1 from bacteria have been made in our lab to determine the methylation activity of Set1 in the absence of other COMPASS proteins. In addition, other members of COMPASS were expressed and purified to reconstitute a minimal COMPASS complex. However, no methyl transfer activity was detected in methylation reactions using bacterially expressed Set1 or a minimal complex containing Set1, Bre2 and Swd1 (data not shown). Recent work has shown that COMPASS can be reconstituted from recombinant proteins expressed in insect cells [24]. We expect that future *in vitro* studies using Set1 mutants in a reconstituted COMPASS complex will provide new insights into substrate binding, catalysis of methyl transfers and product release on both histone and non-histone substrates.

## Supporting Information

Table S1 **Yeast strains used in this study.**
(DOCX)

Table S2 **Oligonucleotides used in this study.**
(DOCX)
