# Supplementary material for: Catalytic and Functional Roles of Conserved Amino Acids in the SET Domain of the S. cerevisiae Lysine Methyltransferase Set1
Source: PLoS One. 2013 Mar 1;8(3):e57974. doi: 10.1371/journal.pone.0057974 (PMC3585878; doi:10.1371/journal.pone.0057974)
Supplement: Table S1 — Yeast strains used in this study. (DOCX) [file pone.0057974.s001.docx]

Table S1. Yeast strains used in this study.

| Name | Genotype |
| --- | --- |
| MBY1198 | *MATα ade2∆::hisG his3∆200 leu2∆0 met15∆0 trp1∆63 ura3∆0* Ty1*his3AI*-236 Ty1*ade2AI*-515 |
| MBY2269 | *MATa ade2∆::hisG his3∆200 leu2^-^ (∆0 or ∆1) met15∆0 trp1∆63 ura3-52 set1∆*::*TRP1* Ty1*his3AI*-236 *ADE2::TELVR* |
| MBY2313 | MBY2269 *ura3-52*::*URA3*-pRS406-*SET1* |
| MBY2315 | MBY2269 *ura3-52*::*URA3*-pRS406 |
| MBY2318 | MBY2269 *ura3-52*::*URA3*-pRS406-*set1-R1013H* |
| MBY2337 | MBY2269 *ura3-52*::*URA3*-pRS406-*set1-Y1054F* |
| MBY2338 | MBY2269 *ura3-52*::*URA3*-pRS406-*set1-Y1054A* |
| MBY2339 | MBY2269 *ura3-52*::*URA3*-pRS406-*set1-F1056Y* |
| MBY2341 | MBY2269 *ura3-52*::*URA3*-pRS406-*set1-N1016A* |
| MBY2382 | MBY2269 *ura3-52*::*URA3*-pRS406-*set1-Y967A* |
| MBY2384 | MBY2269 *ura3-52*::*URA3*-pRS406-*set1-Y993A* |
| MBY2385 | MBY2269 *ura3-52*::*URA3*-pRS406-*set1-H1017L* |
| MBY2386 | MBY2269 *ura3-52*::*URA3*-pRS406-*set1-Y967F* |
| MBY2387 | MBY2269 *ura3-52*::*URA3*-pRS406-*set1-G951A* |
| MBY2396 | MBY2269 *ura3-52*::*URA3*-pRS406-*set1-H1017A* |
| MBY2397 | MBY2269 *ura3-52*::*URA3*-pRS406-*set1-Y1052F* |
| MBY2398 | MBY2269 *ura3-52*::*URA3*-pRS406-*set1-Y1052A* |
| MBY2399 | MBY2269 *ura3-52*::*URA3*-pRS406-*set1-Y1052V* |
| MBY2408 | MBY2269 *ura3-52*::*URA3*-pRS406-*set1-F1056A* |
| MBY2413 | MBY2269 *ura3-52*::*URA3*-pRS406-*set1-H1017R* |
| ZK2 *∆set1* | *MATa ade2^-^ his3∆200 ura3-52 ipl1-2 set1∆::KANMX4* (MBY2450) |
| MBY2511 | MBY2450 *ura3-52::URA3-*pRS406*-SET1* |
| MBY2512 | MBY2450 *ura3-52::URA3-*pRS406 |
| MBY2513 | MBY2450 *ura3-52::URA3-*pRS406*-set1-G951A* |
| MBY2514 | MBY2450 *ura3-52::URA3-*pRS406*-set1-Y967A* |
| MBY2516 | MBY2450 *ura3-52::URA3-*pRS406*-set1-R1013H* |
| MBY2517 | MBY2450 *ura3-52::URA3-*pRS406*-set1-N1016A* |
| MBY2518 | MBY2450 *ura3-52::URA3-*pRS406*-set1-H1017L* |
| MBY2519 | MBY2450 *ura3-52::URA3-*pRS406*-set1-H1017R* |
| MBY2522 | MBY2450 *ura3-52::URA3-*pRS406*-set1-Y993A* |
| MBY2523 | MBY2450 *ura3-52::URA3-*pRS406*-set1-Y1054A* |
| MBY2528 | MBY2450 *ura3-52::URA3-*pRS406*-set1-Y1052V* |
| MBY2529 | MBY2450 *ura3-52::URA3-*pRS406*-set1-F1056A* |
| MBY2530 | MBY2450 *ura3-52::URA3-*pRS406*-set1-F1056Y* |
| MBY2551 | MBY2450 *ura3-52::URA3-*pRS406*-set1-Y967F* |
| MBY2554 | MBY2450 *ura3-52::URA3-*pRS406*-set1-H1017A* |
| MBY2559 | MBY2450 *ura3-52::URA3-*pRS406*-set1-Y1052A* |
| MBY2560 | MBY2450 *ura3-52::URA3-*pRS406*-set1-Y1052F* |
| MBY2561 | MBY2450 *ura3-52::URA3-*pRS406*-set1-Y1054F* |
