# Supplementary material for: Catalytic and Functional Roles of Conserved Amino Acids in the SET Domain of the S. cerevisiae Lysine Methyltransferase Set1
Source: PLoS One. 2013 Mar 1;8(3):e57974. doi: 10.1371/journal.pone.0057974 (PMC3585878; doi:10.1371/journal.pone.0057974)
Supplement: Table S2 — Oligonucleotides used in this study. (DOCX) [file pone.0057974.s002.docx]

Table S2. Oligonucleotides used in this study.

| **Primer** | **Description** | **Sequence** |
| --- | --- | --- |
| OM486 | N1016A sense | ggtggtatagcccgtttcattgctcattgttgtgatccaaattgtacggc |
| OM487 | N1016A antisense | gccgtacaatttggatcacaacaatgagcaatgaaacgggctataccacc |
| OM504 | G951A sense | CGTTCAGCAATTCACAACTGGGCTTTATATGCTCTAGACTCTATCGC |
| OM505 | G951A antisense | GCGATAGAGTCTAGAGCATATAAAGCCCAGTTGTGAATTGCTGAACG |
| OM506 | Y967A sense | GCAAAGGAAATGATTATCGAGGCCGTTGGTGAAAGGATCAGGC |
| OM507 | Y967A antisense | GCCTGATCCTTTCACCAACGGCCTCGATAATCATTTCCTTTGC |
| OM510 | Y993A sense | GGGATTGGATCCAGTGCCCTTTTTAGGGTTGATGAAAACACGG |
| OM511 | Y993A antisense | CCGTGTTTTCATCAACCCTAAAAAGGGCACTGGATCCAATCCC |
| OM512 | H1017L sense | GGTATAGCCCGTTTCATTAATCTTTGTTGTGATCCAAATTGTACGGC |
| OM513 | H1017L antisense | GCCGTACAATTTGGATCACAACAAAGATTAATGAAACGGGCTATACC |
| OM638 | R1013H sense | GCCACCAAGAAAGGTGGTATAGCCCATTTCATTAATCATTGTTGTGATCCA |
| OM639 | R1013H antisense | TGGATCACAACAATGATTAATGAAATGGGCTATACCACCTTTCTTGGTGGC |
| OM708 | Cla1 primer | GGTTTCTTGAACTGACGATGATACATCGATTTGTTTGGAGCTTCCTG |
| OM709 | Cla1 primer | CAGGAAGCTCCAAACAAATCGATGTATCATCGTCAGTTCAAGAAACC |
| OM749 | Y967F sense | GGAAATGATTATCGAGTTCGTTGGTGAAAGGATCAGG |
| OM750 | Y967F antisense | CCTGATCCTTTCACCAACGAACTCGATAATCATTTCC |
| OM751 | Y1054F sense | GCGAAGAGTTGACATATGATTTCAAATTTGAGAGAGAAAAGG |
| OM752 | Y1054F antisense | CCTTTTCTCTCTCAAATTTGAAATCATATGTCAACTCTTCGC |
| OM755 | Y1054A sense | GAAGAGTTGACATATGATGCCAAATTTGAGAGAGAAAAG |
| OM756 | Y1054A antisense | CTTTTCTCTCTCAAATTTGGCATCATATGTCAACTCTTC |
| OM757 | F1056Y sense | GACATATGATTACAAATATGAGAGAGAAAAGGATGACGAG |
| OM758 | F1056Y antisense | CTCGTCATCCTTTTCTCTCTCATATTTGTAATCATATGTC |
| OM851 | Mun1 linker | tgatgccatgaattcgtagcaattggaattctaggtgcat |
| OM852 | Mun1 linker | atgcacctagaattccaattgctacgaattcatggcatca |
| OM947 | F1056A sense | GAAGAGTTGACATATGATTACAAAGCTGAGAGAGAAAAGGATGACGAG |
| OM948 | F1056A antisense | CTTCTCAACTGTATACTAATGTTTCGACTCTCTCTTTTCCTACTGCTC |
| OM951 | H1017A sense | GCCCGTTTCATTAATGCTTGTTGTGATCCAAATTGTACG |
| OM952 | H1017A antisense | CGTACAATTTGGATCACAACAAGCATTAATGAAACGGGC |
| OM953 | H1017R sense | GCCCGTTTCATTAATCGTTGTTGTGATCCAAATTGTACG |
| OM954 | H1017R antisense | CGTACAATTTGGATCACAACAACGATTAATGAAACGGGC |
| OM955 | Y1052F sense | GCGGCAAGCGAAGAGTTGACATTTGATTACAAATTTGAGAGAG |
| OM956 | Y1052F antisense | CTCTCTCAAATTTGTAATCAAATGTCAACTCTTCGCTTGCCGC |
| OM957 | Y1052A sense | GCGGCAAGCGAAGAGTTGACAGCTGATTACAAATTTGAGAG |
| OM958 | Y1052A antisense | CTCTCAAATTTGTAATCAGCTGTCAACTCTTCGCTTGCCGC |
| OM959 | Y1052V sense | GCGGCAAGCGAAGAGTTGACAGTTGATTACAAATTTGAGAG |
| OM960 | Y1052V antisense | CTCTCAAATTTGTAATCAACTGTCAACTCTTCGCTTGCCGC |
